# Volatile Composition, Colour, and Sensory Quality of  Spirit-Based Beverages Enriched with Medicinal Fungus *Ganoderma lucidum* and Herbal Extract

**DOI:** 10.17113/ftb.57.03.19.6106

**Published:** 2019-09

**Authors:** Sonja P. Veljović, Nikola S. Tomić, Miona M. Belović, Ninoslav J. Nikićević, Predrag V. Vukosavljević, Miomir P. Nikšić, Vele V. Tešević

**Affiliations:** 1 Institute of General and Physical Chemistry, University of Belgrade P.O. Box 551, 11001 Belgrade, Serbia; 2 Department for Food Technology and Biochemistry, Faculty of Agriculture, University of Belgrade, Nemanjina 6, 11080 Belgrade, Serbia; 3 Institute of Food Technology, University of Novi Sad, Bulevar cara Lazara 1, 21000 Novi Sad, Serbia; 4 Faculty of Chemistry, University of Belgrade, Studentski trg 16, 11000 Belgrade, Serbia

**Keywords:** aromatic profile, *Ganoderma lucidum*, spirits, GC-MS, herbal extract

## Abstract

The multicomponent mixtures consisting of herbs and fungi are commonly used for the production of alcoholic beverages with potential health-promoting effects in many Asian countries. The medicinal fungus *Ganoderma lucidum* is one of the most important fungi used for spirit production. Although this fungus affects the aromatic complexity of spirits, only a small number of studies have focused on investigating the influence of *G. lucidum* on the aromatic profile and colour of spirits. The aim of the research is to evaluate the influence of adding *G. lucidum* and herbal extract on final concentrations of volatile compounds and sensory quality of several distillates. In this study, distillates (grain, plum, grape and wine) were used to produce new spirit-based beverages with the fungus *G. lucidum* only, or with the fungus and herbal extract. Fifty-nine aroma compounds were identified by GC-MS. The aromatic profiles were strongly influenced by the primary aromas of the distillates, but the addition of *G. lucidum* and herbal extract enriched the volatile fraction of distillates with a range of ethyl esters, with a fruity and floral fragrance. Higher alcohols, 1-propanol, 2-isobutanol and isoamyl alcohol, were the most abundant volatile compounds in the analyzed distillates and spirits. The lightness of distillates was from 60.7 to 63.6, and with the addition of *Ganoderma* it significantly decreased to the range from 43.6 to 50.5. The addition of the fungus also increased the intensity of red and yellow colours. The *Ganoderma* spirits scored very highly in sensory evaluation (17.6–18.3), significantly better than the spirits without any additions (16.1–16.9).

## INTRODUCTION

Spirits are primarily a means of enjoyment and, as such, are commonly consumed due to their pleasant sensory characteristics and the relaxing effect of ethanol. The selection of raw materials used for spirit production encompasses a vast range of cultivated or wild plants worldwide, but strongly depends on local or regional tradition. The raw materials have an important influence on the physicochemical characteristics of spirit-based beverages (*1*, *2*). Primary aromatic compounds which originate from the raw materials are the most important contributors to the authenticity and uniqueness of an alcoholic spirit (*3*, *4*).

In China and other countries of Asia, numerous fungi have been traditionally used to produce alcoholic beverages with potential health-promoting, disease preventing and medicinal qualities. However, multicomponent mixtures consisting of herbs and fungi are most commonly used for the production of such beverages (*5*). It is well established that the medicinal fungi *Ganoderma lucidum* and *Trametes versicolor* are very interesting raw materials for the production of spirits, and they are also rich sources of bioactive compounds (*6*–*8*). In the production of spirits, *G. lucidum* is appreciated because of a specific bitter taste which comes from the bioactive compounds, mainly triterpenoids (*9*). Besides these, *G. lucidum* is a source of volatile compounds that contribute to the aroma of the spirits. Hence, Chen *et al.* (*10*) and Taşkın *et al*. (*11*) conducted studies on *G. lucidum* mycelia and fruiting bodies to analyze their volatile compounds. The former detected 58 compounds in the mycelia of *G. lucidum*, and the predominant volatiles were 1-octen-3-ol, ethanol, hexanal, 1-hexanol, sesquirosefuran, 3-octanol and 3-octanone, while the latter analyzed the volatile aroma compounds of *G. lucidum* collected in the province of Mersin (Turkey) during 2010–2011 and identified 18 aroma compounds, the main being the alcohols 1-octene-3-ol, 3-octanol, 1-octanol, 2-ethyl-1-hexanol, which accounted for about 48% of the compounds responsible for the flavour. Furthermore, another study investigated the volatile compounds of the essential oil fraction produced by *G. lucidum* fruiting body (*12*). The analyzed essential oils consisted of 65 volatile constituents, the major ones being (in %): *trans*- -anethole 9.1, R-(–)-linalool 4.4, S-(+)-carvone 4.4, 2-pentylfuran 2.8, α-terpineol 2.7 and *n*-nonanal 2.3.

*Ganoderma* spirits are commercial products available in local markets of many Asian countries. Until recently, these products have mostly been produced in Asia, but lately, the market expansion has been oriented towards western markets. The aromatic complexity of all spirits, including those enriched with fungus and herbal extract, is an essential parameter of their sensory quality and market positioning. Furthermore, the addition of fungi and herbal extract also changes the colour of alcoholic beverages. To date, only a small number of studies have focused on investigating the influence of fungi, such as *G. lucidum*, on the aromatic profile and colour of spirits.

The aim of this study is to evaluate the influence of adding *G. lucidum* and herbal extracts on final concentrations of volatile compounds and sensory quality of selected distillates produced from different raw materials. Principal component analysis (PCA) was employed to identify the specific compounds with the highest influence on the sensory quality depending on the type of used distillate.

## MATERIALS AND METHODS

### Fungus

*Ganoderma lucidum* (W. Curt.: Fr.) P. Karst fruiting bodies were obtained from Jiangsu Alphay Bio-Tech Co. Ltd. (Nantong, PR China). Fruiting bodies were separated from spores using brushes and then air-dried at 40 °C to constant mass. The fruiting bodies were prepared for extraction by cutting into pieces (about 1 cm).

### Distillates

Different distillates were used to produce new spirit-based beverages with only the fungus *G. lucidum* and fungus plus herbal extract. The following distillates were used: grain distillate produced by the company Ada Vrenje, Belgrade, Serbia; homemade plum distillate from the Aleksandrovac region of Serbia; grape distillate produced at the Radmilovac Experimental School Estate, Faculty of Agriculture, University of Belgrade, Serbia; and wine distillate, an industrial product from Vršački Vinogradi, Vršac, Serbia. All distillates were diluted with distilled water to reach an alcohol strength of 45% (*V*/*V*), chemically analyzed and then used to prepare the spirits with *Ganoderma*.

### Herbal extract

The 44 plant materials (aromatic and medicinal herbs, berries, dried fruits, seeds and tree leaves and bark) were purchased from the Institute for Medicinal Plant Research Dr Josif Pančić, Belgrade, Serbia (Table 1 (*13*)). Altogether, over 10 days, 118.7 g of the plant materials were extracted with 50% (*V*/*V*) ethanol using a shaker (MaxQ 3000; Termo Fisher Scientific, Waltham, MA, USA) at 200 rpm and room temperature ((20±2) °C) in the dark. After extraction, the mixture was pressed with hydraulic press (Atlas Manual Hydraulic Presse, Specac Ltd., Orpington, UK) to separate the liquid from the solids. The obtained liquid was filtered through filter paper (80 g/m^2^, grade 293; Sartorius Stedim Biotech, Vienna, Austria) and stored in a dark place.

**Table 1:** The plant materials used for herbal extract production (13)

Plant	Botanical name	Plant part used	Characteristic
Woodruff	*Galium odoratum* L.	whole plant	bitter/aromatic
Peppermint	*Mentha piperita* L.	leaf	aromatic
Mountain germander	*Teucrium montanum* L.	whole plant	–
Wall germander	*Teucrium chamaedrys* L.	whole plant	bitter
Hibiscus	*Hibiscus rosa-sinensis*	flower	–
Pot marigold or Scotch marigold	*Calendula officinalis* L	flower	aromatic
Stinging nettle	*Urtica dioica* L.	leaf	–
Common sage	*Salvia officinalis* L.	leaf	bitter/aromatic
Chamomile	*Matricaria chamomilla* L.	flower	aromatic
Mellissa	*Melissa officinalis* L.	leaf	aromatic
Sweet flag	*Acorus calamus* L.	root	bitter/aromatic
Wild thyme or creeping thyme	*Thymus serpyllum* L.	whole plant	–
White horehound	*Marrubium vulgare* L.	whole plant	bitter/aromatic
Hawthorn	*Crataegus oxyacantha* L.	flower, leaf	–
Elder or elderberry	*Sambucus nigra* L.	flower	–
Common gypsyweed or heath speedwell	*Veronica officinalis* L.	whole plant	bitter
Common yarrow	*Achillea millefolium* L.	flower	bitter/aromatic
Sweet marjoram	*Origanum majorana* L	whole plant	aromatic
Coltsfoot	*Tussilago farfara* L.	whole plant	–
Common wormwood	*Artemisia absinthium* L.	leaf	bitter
Cypress spurge	*Euphorbia cyparissias* L.	whole plant	–
Field horsetail	*Equisetum arvense* L.	whole plant	–
Common juniper	*Juniperus communis* L	fruit body	bitter/aromatic
Hyssop	*Hyssopus officinalis* L.	leaf, flower	bitter/aromatic
Rosemary	*Rosmarinus officinalis* L.	leaf	aromatic
Gentian	*Gentiana lutea* L.	root	bitter/aromatic
European mistletoe	*Viscum album* L.	whole plant	–
Shepherd’s purse	*Capsella bursa-pastoris* L.	whole plant	–
Herb-paris	*Paris quadrifolia* L.	whole plant	–
European centaury	*Erythraea centaurium* Pers.	whole plant	bitter
Fennel	*Foeniculum vulgare* Mill.	seed	aromatic
Elecampane or horse-heal	*Inula helenium* L.	root	bitter/aromatic
Common chicory	*Cichorium intybus* L.	root	–
Anise	*Pimpinella anisum* L.	seed	aromatic
Ribwort plantain	*Plantago lanceolata* L.	leaf	–
Flat-leaved vanilla	*Vanilla planifolia* Jacks ex Andrews	–	–
Cinnamon	*Cinnamomum* sp.	flower	aromatic
Clove	*Eugenia caryophyllata* L.	bud	aromatic
Fruit	Latin name	Plant part used	Characteristic
Common fig	*Ficus carica* L.	fruit	
Grape	*Vitis vinifera* L.	fruit	
Blueberry	*Vaccinium myrtillus* L.	fruit	
Apple	*Malus domestica* L.	fruit	
Plum	*Prunus domestica* L.	fruit	
Wood	Latin name	Plant part used	Characteristic
Oak wood	*Quercus* sp.	bark	aromatic

### Spirit-based beverage preparation

**Fig. 1:**
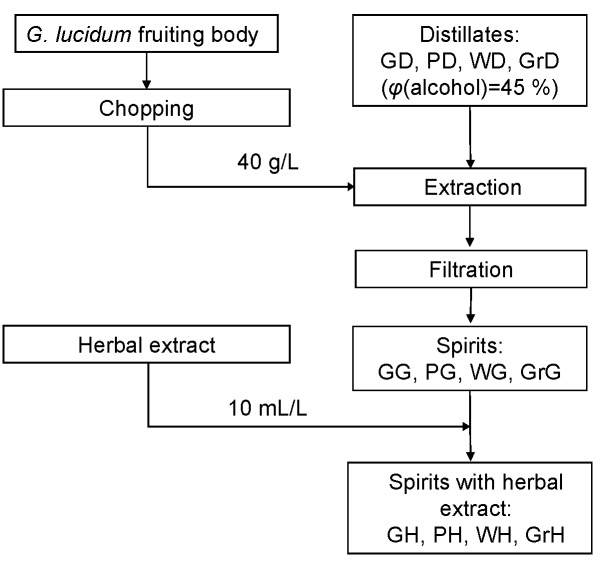
The process of the production of spirits with Ganoderma lucidum and herbal extract. GD, PD, WD and GrD = grain, plum, wine and grape distilate respectively; GG, PG, WG and GrG = grain, plum, wine and grape spirit with G. lucidum respectively; GH, PH, WH and GrH = respective Ganoderma spirits with herbal extract

The production process of spirits with *Ganoderma* is presented in Fig. 1. The chopped *G. lucidum* (40 g/L) was added to the distillates: grain distillate (GD), plum distillate (PD), wine distillate (WD), and grape distillate (GrD). Extraction was performed using a shaker (MaxQ 3000; Termo Fisher Scientific) at 200 rpm and room temperature ((20±2) °C) in a dark place for 60 days. After the extraction, the products were filtered through a filter paper (80 g/m^2^; Sartorius Stedim Biotech), and the obtained *Ganoderma* spirits were further analyzed. Then, 10 mL of herbal extract were added per L of each *Ganoderma* spirit. The types of herbs and their mass per volume ratios for herbal spirit production were chosen according to our previous experience (*14*). All spirits were produced in triplicate.

### Sample preparation for GC-MS analyses

All samples of spirits were prepared using liquid-liquid extraction. An aliquot of 50 mL of each spirit was diluted with 100 mL of distilled water and then mixed with 20 mL of internal standard solution (methyl 10-undecanoate diluted in methylene chloride (0.01 mg/mL)) and 10 g of NaCl. Each resultant mixture was stirred on a magnetic stirrer (MMS 3000; Boeco, Hamburg, Germany) in closed flasks for one hour. After mixing, the organic layer was separated from the water layer. The upper organic layer was dried with Na_2_SO_4_, and then filtered and evaporated on a vacuum evaporator (Hei-VAP Value rotary evaporator; Heidolph Instruments GmbH & CO. KG, Schwabach, Germany) at 10 kPa and 45 °C to a volume of 2 mL. The samples were stored in a refrigerator (4 °C) until analysis.

### GC-MS analyses

Analysis of volatile compounds using GC-MS was performed according to method described by Tešević *et al*. (*4*) with some modifications. The volumes of 1 µL of prepared spirits were injected into the GC system Agilent 7890A (Santa Clara, CA, USA) equipped with 5975C inert XL EI/CI MSD and a FID detector connected by capillary flow technology 2-way splitter with makeup gas. Mass selective detector (MSD) operated in the positive ion electron impact (EI) mode. The separation was achieved on an Agilent 19091N-113 HP-INNOWax fused silica capillary column, 30 m×320 μm×0.25 μm film thickness. The GC oven temperature was programmed from 40 to 220 °C at a rate of 3 °C/min. Helium was used as the carrier gas, the inlet pressure was 25 kPa, and the velocity was 50.4 mL/min at 220 °C. The injector temperature was 250 °C and the injection mode was splitless. The MS scan conditions were source temperature 200 °C, interface temperature 250 °C, and electron beam energy 70 eV. The full-scan mass covered the *m*/*z* range from 40 to 350 atomic mass units (amu).

The identification was performed by comparing the mass spectrum of a compound with the Wiley 275 Mass Spectral Library (*15*) and comparing the resonance ionization (RI) with those available in NIST Standard Reference Data Program 1A (*16*). For quantitative evaluation, the internal standard method was applied, with a known amount of methyl 10-undecanoate as an internal standard (IS).

Semi-quantitative analysis was used to analyze the volatiles in spirit-based beverages. An IS solution (20 mL, 0.01 mg/mL methyl 10-undecanoate diluted in methylene chloride) was added to the sample prior to liquid-liquid extraction. Flame ionization detector (FID, model G1531A; Agilent Technologies) was used for the integrations of all chromatogram peaks. The semi-quantitative concentrations of volatiles in the samples were calculated as follows:

Semiquantitative concentration=((Peak area)_S_/ (Peak area)_IS_)∙*γ*(IS) /1/

where S is a sample and IS is internal standard.

The results of GC-MS analyses were expressed in milligrams of methyl 10-undecanoate equivalents per litre of analyzed spirits.

### Sensory evaluation

Sensory quality rating was conducted in triplicate by a sensory panel that consisted of five expert judges, staff members of the Faculty of Agriculture, University of Belgrade, who were experienced in judging the quality of alcoholic beverages. All experts were male and their age profile ranged from 30 to 60. Three 2-hour training sessions were performed over a period of two weeks using the experimental spirits. Overall sensory quality was assessed by evaluating five selected sensory characteristics: colour, clarity, distinction, odour (oronasal olfaction) and flavour (*4*), which were rated using category scales with score ranges 0–1, 0–1, 0–2, 0–6 and 0–10, respectively. The clarity defines the overall visual liking of beverage; it depends on the purity and colour hue. The distinction clearly deﬁnes speciﬁc and distinctive characteristics typical for certain categories of strong alcoholic beverages. The quality of the spirits was ranked according to the following: excellent quality (quality score>18), very good quality (16<score<18), good quality (14<score<16), poor/unsatisfactory quality (12<score<14), very poor quality (score<12). The overall quality score, with a maximum score of 20, was calculated by adding the quality scores of the five individual characteristics. The spirits were presented to the judges monadically in random order. The panel evaluated all of the spirits once during each sensory evaluation session.

### CIE L*a*b* chromatic parameters

Colour measurements of brandies were obtained using a portable tristimulus Chroma Meter model CR–400 (Konica Minolta, Osaka, Japan) according to the procedure described by Pecić *et al*. (*3*). Results were expressed in CIE L*a*b* chromatic parameters, which are defined by three chromaticity coordinates: L* (lightness), a* (red/green colour component) and b* (yellow/blue colour component).

### Statistical analyses

Sensory evaluation and colour measurements were conducted in triplicate. The analytical colour data and chemical data were subjected to one-way analysis of variance (ANOVA). Sensory data were analyzed by 3-way ANOVA that included ‘brandies’ as fixed factor, and ‘judges’ and ‘replications’ as random factors. Tukey’s HSD test was used to compare the mean values, with the level of statistical significance set at 0.05. Principal component analysis (PCA) on the correlation matrix was applied to raw GC, analytical colour, and sensory data, which had been standardized and averaged. The results for six identified groups of chemical compounds (alcohols, ketones, aldehydes, esters, acids, terpenes and phenols) were summed up to obtain GC data for individual volatiles, with a representative compound chosen from each group. All statistical analyses were performed using Statistica v. 12 (*17*).

## RESULTS AND DISCUSSION

### Aromatic profiles of spirits with Ganoderma

Table 2 shows the volatile fractions identified in the analyzed spirits. The composition of plum distillate volatiles was more complex than of the other spirits. Some specific aromatic compounds were only detected in the plum distillate: nonanol, γ-undecalactone, eugenol and dodecanoic acid. Studies of the volatile compounds of different plum species have confirmed that alcohol (nonanol), fatty acid (dodecanoic acid), phenol (eugenol) and lactone (γ-undecalactone) originated from plum fruit (*18*). The concentration of eugenol in the analyzed plum distillate was lower than in the aged Serbian plum brandies (*4*), which is due to the fact that during ageing, eugenol migrates from the wooden cask into the distillate (*19*).

**Table 2:** Concentrations of some volatile compounds expressed in milligram of methyl-10-undecanoate equivalents per litre of analyzed distillates and spirits

Compound	*γ*/(mg/L)													
		CAS	GD	PD	WD	GrD	GG	PG	WG	GrG	GH	PH	WH	GrH
Alcohols														
1-propanol		71-23-8	0.08^a^	40.35	9.58^b^	7.06^bc^	0.90^a^	2.06^a^	24.81^d^	8.67^bc^	1.00^a^	25.93^d^	3.60^ac^	5.84^bc^
2-methyl-1-propanol		78-83-1	3.55^a^	35.73^d^	49.88^e^	26.99^c^	13.56^b^	16.80^b^	27.36^cd^	31.62	10.81^b^	22.89^c^	n.d.	22.79^c^
1-butanol		71-36-3	0.12^b^	1.81^b^	1.36^b^	1.04^b^	0.03^a^	0.50^b^	1.46^b^	1.23^b^	n.d.	1.19^b^	25.64^c^	0.89^b^
3-pentene-2-ol		1576-96-1	n.d.	0.59^c^	0.63^c^	0.51^bc^	0.54^c^	0.54^c^	0.51^bc^	0.60^c^	0.39^b^	0.53^bc^	0.11^a^	0.43^b^
3-methyl-1-butanol		123-51-3	5.83^a^	99.73^c^	220.98^e^	164.61^d^	3.72^a^	105.20^c^	89.08^c^	186.17^d^	3.13^a^	63.75^b^	0.25^a^	0.03^a^
1-pentanol		71-41-0	0.13	0.19	0.25	0.24	n.d.	0.08	n.d.	0.25	n.d.	0.13	n.d.	0.21
1-hexanol		111-27-3	n.d.	n.d.	n.d.	3.25^b^	n.d.	0.73^a^	0.64^a^	3.63^b^	n.d.	0.48^a^	0.25^a^	2.98^b^
3-methyl-pentanol		77-74-7	n.d.	0.71^a^	1.36^b^	n.d.	n.d.	n.d.	n.d.	n.d.	n.d.	n.d.	n.d.	n.d.
1-decanol		112-30-1	n.d.	n.d.	n.d.	n.d.	n.d.	n.d.	0.20^b^	0.05^a^	n.d.	0.18^b^	n.d.	0.07^a^
benzyl alcohol		100-51-6	n.d.	3.36^d^	n.d.	0.06^a^	n.d.	n.d.	2.83^c^	n.d.	0.09^a^	2.26^b^	0.16^a^	0.13^a^
Ketones														
acetone		67-64-1	n.d.	0.01^a^	0.80^b^	0.48^a^	0.08^a^	n.d.	n.d.	n.d.	n.d.	1.31^b^	n.d.	5.42^c^
2-pentanone		107-87-9	n.d.	0.17^e^	n.d.	0.06^b^	0.01^a^	0.06^b^	0.15^d^	0.06^b^	n.d.	0.11^c^	n.d.	n.d.
4-hydroxy-4-methyl-2-pentanone		123-42-2	0.40	0.25	n.d.	n.d.	n.d.	n.d.	n.d.	n.d.	n.d.	n.d.	n.d.	n.d.
4-methyl-2-pentanone		108-10-1	0.12	n.d	n.d	n.d.	n.d.	n.d.	n.d.	n.d.	n.d.	n.d.	n.d.	n.d.
Aldehydes														
acetaldehyde		75-07-0	n.d.	0.03^a^	0.53^d^	n.d.	n.d.	0.21^b^	0.02^a^	0.33^c^	0.09^a^	n.d.	0.30^c^	0.07^a^
1-hexanal		66-25-1	n.d.	n.d.	n.d.	n.d.	n.d.	n.d.	n.d.	n.d.	n.d.	0.06^b^	n.d.	0.03^a^
benzaldehyde		100-52-7	n.d.	1.82^c^	n.d.	0.11^a^	n.d.	0.05^a^	1.85^c^	0.11^a^	0.03^a^	1.25^b^	0.19^a^	0.14^a^
furfural		98-01-1	n.d.	1.54^c^	n.d.	0.19^a^	0.14^a^	0.35^a^	1.45^c^	0.31^a^	0.15^a^	1.09^b^	0.31^a^	0.26^a^
Esters														
methyl linoleate		112-63-0	n.d.	n.d.	0.12^a^	3.34^b^	3.46^b^	0.11^a^	0.24^a^	6.41^c^	3.25^b^	n.d.	0.47^a^	n.d.
methyl linolenate		301-00-8	n.d.	n.d.	n.d.	0.04^a^	n.d.	n.d.	n.d.	n.d.	n.d.	3.03^b^	n.d.	3.84^c^
methyl salicylate		119-36-8	n.d.	0.08^b^	n.d.	0.05^a^	n.d.	n.d.	0.06^a^	n.d.	n.d.	0.08^b^	n.d.	n.d.
ethyl acetate		141-78-6	5.52^a^	69.52^e^	26.28^b^	28.01^b^	0.08^a^	3.89^a^	58.22^d^	26.23^b^	n.d.	0.04^a^	36.69^c^	0.01^a^
ethyl lactate		97-64-3	n.d.	16.89^d^	4.44^b^	2.00^a^	0.02^a^	1.99^a^	15.28^d^	2.27^a^	0.17^a^	11.64^c^	0.17^a^	1.88^a^
ethyl hexanoate		123-66-0	0.02^a^	n.d.	0.24^b^	0.44^c^	n.d.	n.d.	n.d.	0.59^d^	0.03^a^	n.d.	n.d.	n.d.
ethyl octanoate		106-32-1	n.d.	0.57^b^	0.37^ab^	1.26^c^	n.d.	0.04^a^	0.57^b^	1.73^d^	0.02^a^	0.41^b^	n.d.	0.43^b^
ethyl decanoate		110-38-3	n.d.	0.48^a^	0.27^a^	n.d.	n.d.	n.d.	0.49^a^	1.66^b^	0.13^a^	0.19^a^	n.d.	0.61^b^
ethyl dodecanoate		106-33-2	n.d.	n.d.	n.d.	0.11^a^	n.d.	0.12^a^	0.20^a^	0.58^b^	0.17^a^	n.d.	n.d.	n.d.
ethyl tetradecanoate		124-06-1	n.d.	n.d.	n.d.	n.d.	n.d.	n.d.	0.86^b^	0.84^b^	1.00^b^	0.08^a^	n.d.	0.88^b^
ethyl oleate		111-62-6	n.d.	n.d.	0.10^a^	0.09^a^	3.51^d^	0.15^a^	n.d.	n.d.	n.d.	2.27^c^	n.d.	1.38^b^
ethyl stearate		111-61-5	n.d.	n.d.	n.d.	n.d.	n.d.	n.d.	n.d.	n.d.	0.08	n.d.	n.d.	n.d.
ethyl benzoate		93-89-0	n.d.	0.60^d^	n.d.	0.05^a^	n.d.	n.d.	0.54^d^	0.28^b^	n.d.	0.43^c^	n.d.	n.d.
ethyl salicylate		118-61-6	n.d.	0.07^c^	n.d.	0.06^c^	n.d.	0.03^a^	0.06^c^	0.05^b^	n.d.	0.07^a^	n.d.	n.d.
ethyl cinnamate		103-36-6	n.d.	0.07^a^	n.d.	0.28^b^	n.d.	n.d.	0.09^a^	0.38^d^	n.d.	0.08^a^	n.d.	0.34^c^
diethyl succinate		123-25-1	n.d.	1.82^f^	0.70^c^	0.08^a^	n.d.	0.30^b^	1.60^e^	0.03^a^	n.d.	1.25^d^	0.08^a^	0.23^b^
propyl acetate		109-60-4	n.d.	0.33^e^	n.d.	0.06^a^	n.d.	n.d.	0.31^d^	0.07^a^	n.d.	0.08^b^	n.d.	0.19^c^
isoamyl acetate		123-92-2	10.73^b^	0.20^a^	1.24^a^	0.38^a^	n.d.	n.d.	n.d.	n.d.	n.d.	n.d.	0.36^a^	n.d.
Acids														
acetic acid		64-19-7	n.d.	0.10^a^	n.d.	0.10^a^	n.d.	n.d.	1.81^b^	0.35^a^	0.06^a^	2.23^c^	0.14^a^	0.22^a^
hexanoic acid		142-62-1	n.d.	0.43^a^	n.d.	n.d.	n.d.	n.d.	0.55^b^	0.59^b^	n.d.	n.d.	0.37^a^	n.d.
octanoic acid		124-07-2	n.d.	0.83^b^	0.40^b^	0.93^b^	n.d.	n.d.	0.82^b^	1.60^c^	0.08^a^	0.67^b^	4.98^d^	1.80^c^
decanoic acid		334-48-5	n.d.	0.91^b^	n.d.	1.83^c^	0.11^a^	0.15^a^	0.85^b^	2.98^d^	0.17^a^	0.85^b^	n.d.	3.23^d^
dodecanoic acid		143-07-7	n.d.	0.41^a^	n.d.	n.d.	n.d.	n.d.	0.68^ab^	1.42^c^	n.d.	0.79^b^	n.d.	2.54^d^
hexadecanoic acid		57-10-3	n.d.	0.74^ab^	n.d.	0.47^a^	1.22^ab^	0.59^a^	n.d.	n.d.	1.75^b^	1.12^ab^	2.52^ab^	1.26^ab^
oleic acid		112-80-1	n.d.	n.d.	n.d.	n.d.	n.d.	n.d.	n.d.	n.d.	n.d.	n.d.	0.21	n.d.
linoleic acid		60-33-3	n.d.	n.d.	n.d.	n.d.	n.d.	n.d.	n.d.	n.d.	n.d.	n.d.	0.54	n.d.
Terpenes and phenols														
linalool		78-70-6	n.d.	0.10^a^	n.d.	0.45^b^	n.d.	n.d.	0.08^a^	0.44^b^	n.d.	n.d.	n.d.	n.d.
terpinen-4-ol		20126-76-5	n.d.	n.d.	n.d.	n.d.	n.d.	n.d.	n.d.	n.d.	n.d.	0.04^a^	n.d.	0.06^b^
nonanol		143-08-8	n.d.	0.19^a^	n.d.	n.d.	n.d.	n.d.	n.d.	n.d.	n.d.	n.d.	n.d.	0.60^b^
α-terpineol		98-55-5	n.d.	0.20^d^	n.d.	0.14^b^	n.d.	n.d.	0.16^c^	0.12^a^	n.d.	0.24^e^	n.d.	n.d.
eugenol		97-53-0	n.d.	0.47^b^	n.d.	n.d.	n.d.	n.d.	0.50^b^	0.12^a^	0.56^b^	0.88^c^	0.22^a^	0.58^b^
neo-menthol		491-01-0	n.d.	n.d.	n.d.	0.13^a^	n.d.	n.d.	n.d.	n.d.	n.d.	0.48^b^	1.66^c^	0.11^a^
phytol		7541-49-3	n.d.	n.d.	n.d.	n.d.	n.d.	n.d.	0.29^b^	0.40^c^	0.16^a^	n.d.	n.d.	n.d.
spathulenol		6750-60-3	n.d.	n.d.	n.d.	n.d.	n.d.	n.d.	n.d.	n.d.	n.d.	0.07	n.d.	n.d.
vanillin		121-33-5	n.d.	n.d.	n.d.	n.d.	n.d.	n.d.	n.d.	n.d.	n.d.	n.d.	1.40	n.d.
phenyl ethanol		1445-91-6	n.d.	n.d.	4.65^c^	1.30^a^	n.d.	n.d.	n.d.	n.d.	n.d.	2.12^b^	n.d.	n.d.
Miscellaneous														
tridecane		629-50-5	0.21	n.d.	n.d.	n.d.	n.d.	n.d.	n.d.	n.d.	n.d.	n.d.	n.d.	n.d.
1,1-diethoxyetane		105-57-7	n.d.	1.55^ab^	1.24^b^	9.34^d^	2.88^b^	0.12^a^	5.78^c^	10.45^d^	n.d.	0.17^a^	15.15^e^	0.41^ab^
1,1-diethoxyhexane		222-911-4	0.07	n.d.	n.d.	n.d.	n.d.	n.d.	n.d.	n.d.	n.d.	n.d.	n.d.	n.d.
*γ*-undecalactone		104-67-6	n.d.	0.10	n.d.	n.d.	n.d.	n.d.	n.d.	n.d.	n.d.	n.d.	n.d.	n.d.
*p*-menth-3-ene		500-00-5	n.d.	0.06^b^	n.d.	0.13^c^	n.d.	n.d.	0.07^b^	0.13^c^	0.02^a^	0.06^b^	n.d.	0.15^d^

Values marked with the same letter within the same row are not statistically different (α=0.05). Abbreviations are given in Fig. 1

Plum distillate was also different from the other spirits with *Ganoderma* because it had a significantly higher concentrations of ethyl acetate and ethyl lactate (Table 2). Since this distillate is not produced under strictly controlled conditions, the naturally occurring microbiota likely had a more significant impact than in the other, industrially produced, distillates. Ethyl acetate was the most abundant ester, accounting for 76.7% of all esters, which is in agreement with previous studies by Satora and Tuszyński (*20*). The ethyl acetate in homemade plum distillate likely formed during fermentation in the presence of diverse epiphytic microorganisms, including wild yeast and acetic acid bacteria (*20*). Ethyl lactate, with a pleasant fruity fragrance, was another important ester of the plum distillate (Table 2). Higher alcohols, such as 1-propanol, 2-methyl-1-propanol (isobutanol), 3-methyl-1-butanol (isoamyl alcohol), had an important role in the formation of plum distillate aroma (Table 2). The concentration of 1-propanol was higher than the concentration of isobutanol, as a previous study confirmed (*21*).

Wine distillate is a product derived from wine. It contained significantly higher concentrations of higher alcohols than other spirits, in particular isobutanol and isoamyl alcohol (Table 2), as reported by Tsakiris *et al.* (*22*). Phenyl ethanol was the only detected terpene alcohol in the wine distillate (Table 2). The concentration of this volatile compound with floral (rose) nuances was 4.65 mg/L. Phenyl ethanol was detected at the same concentration in grappa, but the concentration was higher in orujo, both spirits produced from grape marc (*23*). Quantitatively, the most abundant ester in our wine distillate was ethyl acetate with a concentration of 26.27 mg/L, followed by ethyl lactate at 4.44 mg/L.

Grape distillate is produced by distillation of fermented grape mash. The initial raw materials for grape and wine distillate production are similar. Thus, they both contain some unique compounds which were detected only in these products, such as ethyl oleate and methyl linoleate (Table 2). However, the grape distillate contained higher concentrations of fatty acids and ethyl esters (Table 2). The concentration of ethyl esters with floral or fruity aromas can be increased during ageing, as a result of the esterification of different organic acids with ethanol (*22*). However, a few studies found that the concentration of ethyl esters can also decrease during ageing due to the interaction between ethanol and water (*24*, *25*). Moreover, the content of esters strongly depends on the grape variety used (*26*).

The volatile fraction of our grain distillate contained only 12 compounds (Table 2), and most of them were produced during fermentation by the enzyme complexes of yeasts. Hydrocarbon compounds (1,1-diethoxyhexane and tridecane) and ketone (4-methyl-2-pentanone) were detected only in the grain distillate (Table 2). Aldehydes and higher alcohols have a negative effect on the aroma of spirit-based beverages (*2*).

The compounds extracted from *G. lucidum*, which were also components of the *Ganoderma* spirits, mainly caused changes in the concentrations of the already existing compounds. However, the alcohol 1-hexanol was an exception since it was detected in all *Ganoderma* spirits, although it was initially present only in the grape distillate. Thus, the amounts of 1-hexanol detected in the grain, wine and plum spirits with *Ganoderma* originated from the fungus (*11*). The concentrations of 1-hexanol in the grain, wine and plum spirits with *Ganoderma* were significantly lower than in the grape spirit with *Ganoderma* (3.63 mg/L; Table 2). Although all distillates initially contained isoamyl acetate, which has a specific banana flavour, it was not detected in the spirits after the addition of the fungus. A significant difference was found in the fatty acid content between the spirits with the fungus and their equivalent distillates before its addition. Accordingly, it can be concluded that the fatty acids were also extracted from the fungus. A previous study found that the essential oil of this fungus is a significant source of terpenes (*12*). Despite that, the addition of fungus did not significantly change the composition of terpenes in the spirits.

During the brandy ageing process, numerous reactions occur in the alcohol-water mixture. Isoamyl alcohol was the dominant compound in all spirits with *Ganoderma*, but its concentration was significantly decreased after the addition of the fungus to the grape distillate. A common compound in alcoholic spirits is also acetal, 1,1-diethoxyethane formed by the reaction of acetaldehyde with ethanol (*27*). Therefore, in all analyzed spirits with fungus, the acetal concentration increased and the pungent odour of aldehydes was reduced compared with the equivalent brandies before the addition of the fungus (*19*).

Eugenol and furfural, characteristic quaternary compounds, were present in all the spirits with *Ganoderma*, but the grain and wine distillates did not contain furfural (Table 2). These compounds were likely extracted from *G. lucidum* and so were detected in the *Ganoderma* spirits. Furthermore, this medicinal fungus has a wood-like structure consisting of similar compounds, such as tannins, as the wood utilized for casks used during normal brandy production (*28*).

Aromatic herbs are traditionally used to enrich the aroma of distillate and form new, sophisticated spirits. Volatile compounds of herbal extracts usually constitute a complex mixture, with each compound individually at low concentration. In this research, the influence of the herbal extract on the volatile fraction of the spirits produced with *G. lucidum* was investigated. Based on the results of GC-MS analyses, the aromatic profile of the analyzed spirits was strongly influenced by the distillate used for their production.

The addition of *G. lucidum* and herbal extract enriches the volatile fraction of spirits with a wide range of ethyl esters that originate from fatty acids, and provide a pleasant fruity and floral fragrance (*29*). Qualitative and quantitative composition of ethyl esters strongly depended on the composition of the initial distillates used as bases. All distillates used for the production of *Ganoderma* spirits had the equal content of ethanol, which had mostly influence on the extraction process. Based on our results, it can be concluded that the other compounds of the alcohol-water mixtures also had an important influence on the solubility of compounds from *G. lucidum* and the herbal extract, and strongly affected the volatile profile and sensory characteristics of *Ganoderma* spirits.

The main chemical compounds detected in spirits enriched with *G. lucidum* and herbal extract were higher alcohols and aldehydes (Table 2). The dominant higher alcohols and aldehydes were different between these distillates, depending on the initial distillate used. In the grain brandy with *G. lucidum* and herbal extract, the higher alcohols 3-methyl-1-butanol (isoamyl alcohol) and 2-methyl-1-propanol (isobutanol) were the most abundant (Table 2). Additionally, this distillate also contained 1-propanol and 2-methyl-1-propanol, which when present at higher concentrations have a penetrating odour and alcohol taste, respectively (*19*).

### Sensory quality of spirits

Sensory quality scores (colour, clarity, distinction (the typical character), odour and flavour) of the distillates and spirits with *Ganoderma lucidum* or with *G. lucidum* and herbal extract are shown in Table 3.

**Table 3:** Sensory quality scores for distillates and spirits produced with the addition of *Ganoderma lucidum* and herbal extract

Sample	Colour	Clarity	Distinction	Odour	Flavour	Overall score
GD	1	1	2	4.5±0.1	7.6±0.2	(16.1±0.1)^a^
PD	1	1	2	5.0±0.2	8.0±0.1	(16.9±0.1)^c^
WD	1	1	2	5.1±0.1	7.4±0.1	(16.5±0.1)^b^
GrD	1	1	2	4.8±0.4	7.5±0.2	(16.5±0.2)^b^
GG	1	1	2	5.5±0.1	8.7±0.2	(18.1±0.1)^de^
PG	1	1	2	5.4±0.2	8.2±0.1	(17.6±0.1)^d^
WG	1	1	2	5.5±0.1	8.7±0.1	(18.2±0.1)^e^
GrG	1	1	2	5.4±0.4	8.5±0.2	(17.8±0.2)^d^
GH	1	1	2	5.4±0.1	8.6±0.2	(18.1±0.1)^de^
PH	1	1	2	5.5±0.2	8.8±0.2	(18.3±0.1)^e^
WH	1	1	2	5.5±0.1	8.7±0.1	(18.2±0.1)^e^
GrH	1	1	2	5.5±0.1	8.6±0.1	(18.1±0.1)^e^

Values are arithmetic mean±standard deviation (5 assessors, 1 replication). Values of overall score marked with the same letter are not statistically different (*α*=0.05). Abbreviations are given in Fig. 1

According to the obtained results, the addition of *G. lucidum* improved some sensory characteristics of the analyzed brandies. The total sensory scores of all *Ganoderma* spirits were in the range from very good to excellent quality (17.6 to 18.3). The *Ganoderma* spirits based on wine or grain distillate received better scores than the *Ganoderma* spirit based on the homemade plum brandy. A distillate with a simple sensory profile is more suitable for the production of *Ganoderma* spirit than more complex bases, such as plum distillate.

The herbal extract was added to the *Ganoderma* spirits as an attempt to enrich their sensory characteristics. The sensory qualities of the spirits with *Ganoderma* based on grape distillate and plum distillate were significantly improved after the addition of the herbal extract. The addition of the herbal extract had a positive effect on the flavour of all spirits with *Ganoderma* (Table 3).

### Colour of spirits

**Fig. 2:**
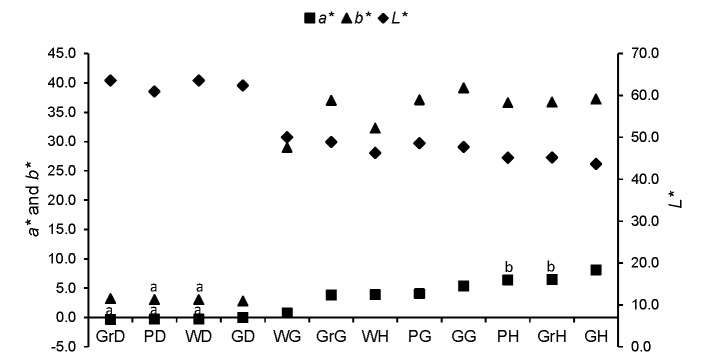
Colour measurement of the spirits in the CIE L*a*b* colour system. Values for a variable marked with the same letter are not statistically different (α=0.05). Standard deviation ranges: L*=0.00–0.02, a*=0.00–0.04, b*=0.00–0.04. Abbreviations are given in Fig. 1

The raw materials used, such as the fungus *G. lucidum* and the herbs and plant materials, significantly contributed to the colours of the spirits. The results of one-way ANOVA indicate that the differences between L*, a* and b* parameters of the analyzed distillates were significant (Fig. 2). The content of soluble compounds in the alcohol-water mixtures used as bases strongly influenced the lightness of the finished spirits. The lightness of plum distillate (60.7) was lower than the other used distillates (62.4–63.6).

The addition of *G. lucidum* and herbal extract to the distillates statistically significantly influenced the L* value, which was in the range from 47.7 to 50.5 for spirits enriched with the fungus, and from 43.6 to 46.3 for spirits enriched with the fungus and the herbal extract. Since the same mass of fungus was used for the production of all spirits, the initial distillate had an important influence on the lightness of the final products. Comparing the current results with our previous research (*9*), it can be concluded that the type of distillate had a more significant effect on the lightness of the produced spirits than the extraction period when the same mass of the fungus was added. The addition of *G. lucidum* and herbal extract decreased the lightness of grain-based spirits to a greater extent than the other used bases, and thus, these brandies had lower L* values than other spirits.

The value of parameter a* was in the range from –0.3 (plum, wine and grape distillates) to 8.1 (grain spirit with *Ganoderma* and herbal extract). The a* values of the distillates (grain, plum and grape) were negative, and it was noted by the panel that these spirits were characterized by light tones of green colour. The addition of fungus and herbal extract increased the intensity of the resultant spirits to amber shades, characteristic of old cognacs. The b* values were positive, and in the range from 2.8 (grain distillate) to 39.2 (grain spirit with *Ganoderma*). Hence, all analyzed spirits had different intensities of yellow colour. Based on these results, it can be concluded that the addition of *G. lucidum* and herbal extract increased the intensity of yellow, from golden to caramel. The L*, a* and b* values were significantly different for the wine distillate with *Ganoderma*, and the sensory experts described its colour as olive green with a shade of yellow as the background.

### Principal component analysis

**Fig. 3:**
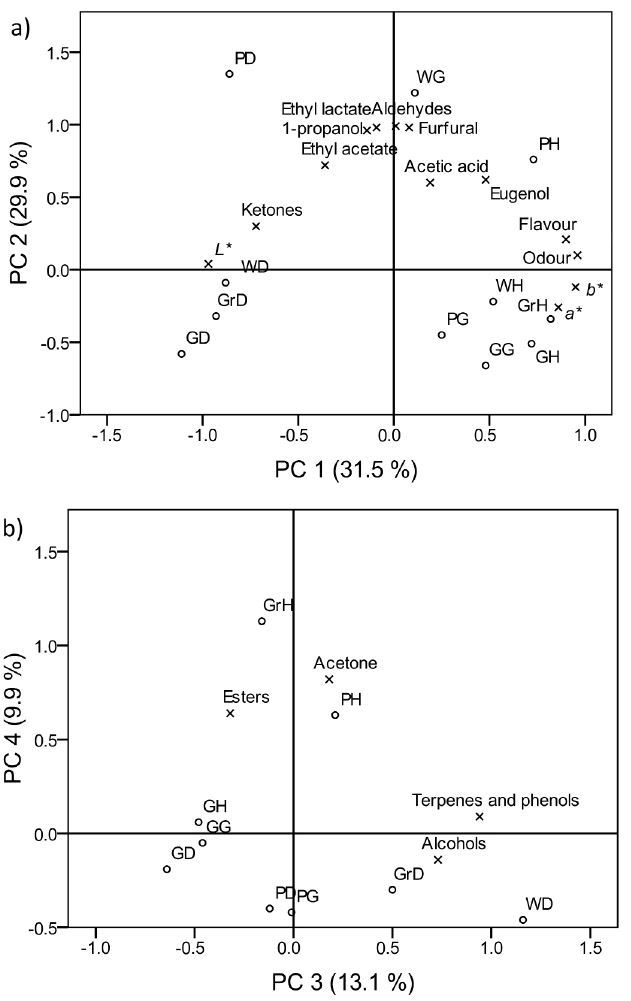
Two biplots of the first four principal components obtained by principal component analysis (varimax rotation) of standardized chemical, colour and sensory (flavour and odour) data of spirits produced with the addition of *Ganoderma lucidum* and herbal extract. Variables with both loadings <0.60 and spirits with both scores <0.40 are suppressed. Abbreviations are given in Fig. 1

PCA was applied in order to assess how the obtained spirits were grouped, taking into account the composition and content of aromatic compounds, colour attributes and sensory characteristics (Fig. 3). PCA was previously shown to be a reliable tool for discrimination between brandy samples according to the composition of volatile compounds (*21*, *30*). In the current study, the first five extracted principal components had eigenvalues larger than 1, but according to the scree plot (eigenvalues stopped decreasing rapidly at the fourth point) and the component matrix (the loading values for PC 5 were all below 0.55), only the first four components were retained, explaining 84.4% of the variance in the data matrix values. Varimax rotation was chosen since it showed the best arrangement of the loading values in comparison with other solutions.

Fig. 3a shows the biplot of loadings and scores of the first two extracted components, while the biplot of the last two components is shown in Fig. 3b. According to the biplots, higher scores for odour and flavour quality were given to the spirits characterized by higher concentrations of esters and acetone (such as grape and plum spirit with *Ganoderma* and herbal extract), and spirits with lower concentrations of ketones (such as all spirits produced with *Ganoderma* and herbal extract). From each group of chemical compounds detected by GC-MS representatives (eugenol, acetic acid, furfural, 1-propanol, ethyl lactate and ethyl acetate) were chosen, and the influence of these volatiles cannot be explained within the obtained four-dimensional PC space. Higher contents of these volatiles were found in plum distillate, wine spirit with *Ganoderma* and plum spirit with *Ganoderma* and herbal extract on the upper side of the PC1–PC2 plot (Fig. 3a). The other identified aldehydes also had the highest content in these spirits. As the biplots show, the most specific spirit with *Ganoderma* was the wine distillate. Only the aromatic profile of this spirit contained vanillin (Table 2), although vanilla was used as part of the plant mixture used for the production of herbal extract. Vanillin has a pleasant aroma and is also one of the characteristic quaternary compounds of aged brandies (*19*). Consequently, wine distillate with *Ganoderma* received the best sensory scores among the *Ganoderma* spirits. It also had a low content of extracted compounds from the fungus that affected its colour. The plum-based spirit with *Ganoderma* and herbal extract had the highest concentration of eugenol with a characteristic aroma of cloves, so was judged positively for the sensory characteristics of flavour and odour. The spirits on the far-right side of the PC1–PC2 plot (Fig. 3a) had more pronounced amber colour with a shade of caramel as a background colour and were darker, while the spirits on the opposite, far left side of the plot (grain, grape, wine and plum distillate) had more pronounced yellow colour and were lighter. Considering the analyzed spirits, the added fungus and herbal extract influenced the lightness, so these brandies were darker and contained more extracted compounds. The wine and grape distillates had the highest contents of alcohols, terpenes and phenols (Fig. 3b).

Spirits based on grain are grouped on the left side of the PC3–PC4 plot (Fig. 3b). The extracted compounds from the fungus and herbs strongly affected the colour of these spirits. An additional difference arose from the similar content of ketones in grain-based spirits.

## CONCLUSIONS

According to the results obtained in the present work, the used distillates had a strong influence on the aromatic profile of the produced spirits. The chemical composition of the distillate bases had an important influence on the solubility of the components originating from *Ganoderma lucidum* and the herbs, and thus determined the sensory characteristic of the final spirits. Owing to the complexity of the material used for spirit production, PCA was employed to examine their influence on volatile composition of the final product. The results of PCA showed significant influence of six identified groups of chemical compounds (alcohols, ketones, aldehydes, esters, acids, and terpenes and phenols) on the sensory quality of the spirits with the fungus. The higher scores for odour and flavour quality were given to the spirits characterized by higher contents of esters and acetone such as grape and plum distillates with *Ganoderma* and herb extract, and lower contents of ketones such as grape, plum, grain and wine distillates with the fungus and herb extract. The addition of *G. lucidum* and herbal extract decreased the lightness of the spirits, and increased the intensity of yellow and red colours compared to the initial distillates. The obtained results have shown that the addition of *G. lucidum* and the herbal extract had a positive effect on the sensory quality of all spirits with *Ganoderma*. *Ganoderma* spirits could be potential innovative products for regional markets.
